# A School Intervention's Impact on Adolescents' Health-Related Knowledge and Behavior

**DOI:** 10.3389/fpubh.2022.822155

**Published:** 2022-03-14

**Authors:** Gabriella Nagy-Pénzes, Ferenc Vincze, Éva Bíró

**Affiliations:** ^1^Department of Public Health and Epidemiology, Faculty of Medicine, University of Debrecen, Debrecen, Hungary; ^2^Doctoral School of Health Sciences, University of Debrecen, Debrecen, Hungary

**Keywords:** adolescent and youth, school health promotion programme, school health promotion and prevention, health education, intervention study, health-related knowledge, health behavior

## Abstract

**Background:**

Many factors can influence health behavior during adolescence, and the lifestyle of adolescents is associated with health behavior during adulthood. Therefore, their behavior can determine not only present, but also later health status.

**Objective:**

We aimed to develop an intervention program to improve high school students' health behavior and to evaluate its effectiveness.

**Methods:**

We performed our study at a secondary school in a rural town in East Hungary between 2016 and 2020. Sessions about healthy lifestyles were organized regularly for the intervention group to improve students' knowledge, to help them acquire the right skills and attitudes, and to shape their behavior accordingly. Data collection was carried out via self-administered, anonymous questionnaires (*n* = 192; boys = 49.5%; girls = 50.5%; age range: 14–16). To determine the intervention-specific effect, we took into account the differences between baseline and post-intervention status, and between the intervention and control groups using individual follow-up data. We used generalized estimating equations to assess the effectiveness of our health promotion program.

**Results:**

Our health promotion program had a positive effect on the students' health-related knowledge and health behavior in the case of unhealthy eating, moderate to vigorous physical activity, and alcohol consumption.

**Conclusion:**

Our findings suggest that school health promotion can be effective in knowledge transfer and lifestyle modification. To achieve a more positive impact on health behavior, preventive actions must use a complex approach during implementation.

## Introduction

Adolescents' health behavior changes with age; however, the vast majority of behaviors and habits acquired at this age persist into adulthood, so these behaviors determine not only present, but also later health status.

The health behavior of Hungarian school-age children is not very favorable in an international context. Hungarian children eat more unhealthily (less frequent breakfast, fruit and vegetables and more frequent sweets and sugared soft-drinks consumption) than their counterparts in other countries, with a higher proportion of regular smokers, drinkers, and those who are sexually active. Hungarian adolescents consider their health to be more unfavorable, and it is more common for them to be overweight and obese (1, 2).

In addition to gender (e.g., girls exercise less, their mental health is less favorable) and age (e.g., older adolescents are more prone to risky behaviors) (1, 2), adolescents' health and health behavior may be affected by their socioeconomic status and social relationships. Teenagers with better family affluence—based on material assets in the household and traveling for a holiday—are more satisfied with their lives and less overweight (1). Healthy eating is more frequent (3), and extreme alcohol consumption is lower when parents are highly educated (4, 5). Students who experience greater social support are more likely to cope with their everyday problems, have better mental health, and are less affected by physical and mental symptoms (6–9).

Today, there is a strong emphasis on mass media, advertising and social media, which can influence an individual's behavior, attitude, and self-image (10–13). Internet orientation and mass media can be very useful for health (14–17), but they can also pose great dangers due to the lack of credibility and social media trends (18).

School also plays an important role in shaping lifestyle because children spend a significant part of their time in school, which is a crucial area for institutional socialization. Therefore, health promotion in school is of paramount importance in promoting adolescents' health behavior and health. School can also play a key role in helping the adolescents filter out misinformation by disseminating and using credible sources of information online and promoting critical thinking.

In 2000, the International Union for Health Promotion and Education (IUHPE) collected and evaluated evidence on the effectiveness of health promotion, including school health promotion, over the previous 20 years. The IUHPE found that interventions were most effective when, together with behavioral change, they: focused on academic and social outcomes; were comprehensive and holistic; linked school to health organizations and other sectors; were sufficiently deep; had been running several school years; and fundamentally influenced students' social and academic growth (19).

Several literature review studies have compared the methods of school interventions and their effectiveness. Evidence suggests that more intensive (20) interventions targeting multiple (risk) behaviors at the same time (21, 22) may be more successful. Relatively few studies have examined the long-term impact of school health promotion programs, but the ones that do exist found that the positive effects of interventions may disappear, so longer-term maintenance of programs or occasional “reminder” sessions may be warranted. Involving parents and the school's partners or local community enhances the effectiveness of interventions (20, 21), but more emphasis should be placed on involving these actors and examining their impact (22, 23). The abovementioned reviews concluded that more, better-designed, reliable intervention studies with long-term follow-up are needed, with the potential to determine effectiveness with greater certainty; hence, it is important to strive for program sustainability (21–24).

The world's largest health and disease prevention organizations—including the US Centre for Disease Prevention and Control, the World Health Organization, and the School for Health in Europe Network Foundation—strongly support the complex approach of school health promotion (25–27).

In Hungary, there are numerous laws (28–31); governmental (32, 33) and non-governmental (34–38) organizations alike have taken public health actions and launched programs to foster adolescents' health and health behavior in the past few years. However, whether these methodologically extremely heterogeneous initiatives have actually improved the health and health behavior of Hungarian adolescents is not clear due to a lack of documented evaluation of their effectiveness. Therefore, we wished to develop a replicable and sustainable health promotion program among high school students and to determine its effectiveness.

## Materials and Methods

### Study Population

We carried out our study at a secondary school in a rural town in East Hungary between 2016 and 2019. We performed the recruitment, inclusion, and randomization of 9th-grade adolescents in two waves in autumn of 2016 and 2017 (in total 12 classes with 260 pupils). The school is a mixture of high, vocational high, and secondary school institution types. From all the attainable classes, we randomly allocated six classes by institution type to the control group, where participants received no intervention, and six classes to the intervention group. We invited all students of the classes to participate in the survey. We sent consent forms to the parents of all adolescents; only students who received parental consent and gave active consent themselves were eligible to be involved. The study was conducted according to the guidelines of the Declaration of Helsinki and approved by the Hungary's Medical Research Council Scientific and Research Committee (49460–5/2016/EKU).

### Theoretical Framework of the Study

To design effective interventions, a team of psychologists synthesized all the theories describing behavioral change and developed the COM-B model, which describes the conditions influencing behavior. In the COM-B model, capability (the physical and psychological ability to enact a behavior), opportunity (the physical and social environment that enables a behavior), and motivation (the reflective and automatic mechanism that activates/inhibits a behavior) all affect an individual's behavior, thereby also influencing the previously listed factors; capability and opportunity separately affect one's motivation (39). We used this model as the theoretical framework for our study: We assumed that by developing students' abilities, enhancing motivation, and changing environmental factors, we could facilitate a positive effect on the health behavior of adolescents and thus on their health status (Table 1).

**Table 1:** Methods designed to promote positive student health behavior and to evaluate their effectiveness in light of the COM-B model.

**Capability**	
**Methods for shaping abilities and skills**	**Evaluation of effectiveness**
**Physical capacity:** The students' physical capacity was taken as given	–
**Psychological capacity: Knowledge and skills development** • Basic biological, anatomical, and physiological knowledge • Describing the advantages, disadvantages, and consequences of health behaviors to make responsible decisions • Authentic sources of information and the use of smartphone applications • Discussing experiences	Knowledge test on intervention topics
**Motivation**	
**Methods used to foster motivation**	**Evaluation of effectiveness**
**Creating intention (reflective processes), attitude-forming:** • Main message: Health is a value that needs to be protected and developed. • Group tasks • Arguments for healthy or risky behaviors and against; budgeting. • Situational practice, playful tasks, tasks using phone applications and websites • Presence of a public health professional or teacher • Encouragement	Questionnaire on health behavior and attitudes
**Habits (automatic processes):** • Strengthening and striving to learn good habits • Striving to stop unfavorable habits	
**Opportunities**	
**The environment that supports the intervention as a resource**	**Evaluation of the environment**
**Social environment:** • School management, classroom teachers, physical education and science teachers • Class community • Parents (who agreed to have their child participate in the study) • The work of the health promotion office	• Family support • Friends' support • Friends' health behavior • Classmates' support • Teacher's support • Attitudes toward school
**Physical environment: School and home** • Difficult to shape: infrastructural constraints; the control group is also in the school. • Classroom decorations for lessons	–
**Behavior**	
**The intervention aims to positively influence the following behaviors**	**Evaluating the intervention's effectiveness**
• Nutrition • Physical activity • Screen time • Smoking • Alcohol consumption • Substance abuse • Sexual behavior	Health behavior survey with questionnaire
**Health status**	
**Physical:** • Chronic illness • Body mass index (BMI) • Body fat percentage **Mental:** • Self-rated health • Life satisfaction • Self-esteem • Depression	**Aim of the intervention** Positively influence nutritional status and mental health **Evaluating the effectiveness:** Hungarian National Student Fitness Test (NETFIT®) data, questionnaire

*Text in gray: We have planned these parts of the intervention study, but we do not discuss them in the manuscript; or we were not able to implement them*.

The basis of our intervention was the development of capability (i.e., provide the necessary knowledge and improving skills) and the enhancement of motivation (through increasing knowledge and understanding, persuasion to elicit appropriate feelings about the behavior and stimulate action) (39). Our intervention did not cover students' physical ability; it was taken as given. We have not measured students' motivation directly but decided to assess their attitudes because a positive attitude could increase motivation, and we thought that this interpretation is closer to the COM-B framework (39) (i.e., the possible ways of motivation enhancement during an intervention). Regarding the students' social environment, the cohesion among classmates was improved through games and group tasks. The management of the school and the teaching staff were supportive and inquiring, but were not involved directly in the intervention. The involvement of the parents was planned, but could not happen due to low parental activity. On the other hand, we were able to contact the staff of the local health promotion office as part of the health sector and to carry out some of our work with them. The shaping of the physical environment was limited by infrastructural constraints and the fact that the members of the control group were also students of the given educational institution, which is why we were able to change the classrooms only for the duration of the sessions with the help of posters and pictures on the blackboard. We also monitored changes in students' physical and mental health. Sessions related to mental health would have received more emphasis in the 2019–2020 school year; however, most of these classes were canceled due to the COVID-19 pandemic (Table 1).

### The Intervention

First, we carried out a baseline survey to assess the students' health-related knowledge, attitudes toward a healthy lifestyle, health behavior, and health status. We planned the health promotion sessions based on the main results of the baseline survey, so the main problems determined the topics.

We organized regular healthy lifestyle sessions for the intervention group. These were embedded into the curriculum (built into lessons of different subjects) and usually required 2–3 lessons per academic year for each topic. Our goal was to expand students' knowledge, to help them acquire the right skills and attitudes, and to shape their behavior accordingly through classes led by a public health professional and a public health student.

We built the sessions around the following topics (in line with the knowledge test and the health behavior questionnaire): health as a value, nutrition, physical activity, sexuality, addiction, alcohol consumption, smoking, substance abuse, cancer in light of lifestyle factors, and mental health. We wanted to include the students in our research until they finished their studies, but some of the intervention sessions and the planned end-of-study surveys were canceled due to the COVID-19 pandemic.

To help the students gain diverse knowledge, skills, attitudes, and behaviors, we employed various interactive methods and tools such as mind maps; individual, pair and group work; presentations; posters; professional websites; and telephone applications.

### Data Collection to Measure and Evaluate the Intervention's Effectiveness

In both the intervention and control groups, all students were assessed *via* an anonymous, self-administered questionnaire at baseline and immediately at the endpoint (post-intervention) of the intervention (2 and 3 school year follow-up) in a cross-sectional manner. All students had a unique code for anonymous individual follow-up and they also had to answer questions like the password reminders for the case if they would forget their code.

With the baseline survey, we wished to determine the participants' baseline characteristics, as well as the possible differences between the intervention and control groups. With the post-intervention survey, we assessed changes in student health-related knowledge (as a proxy of their capability), attitudes toward a healthy lifestyle (related to their motivation), health behavior, and health status that were attributable to the intervention. We also administered the post-intervention survey to the control group to describe the changes among the subjects that were independent of the intervention. All of the questionnaires used at each time point were identical and completed in a classroom setting, supervised by a research team member.

The first part of the questionnaire focused on demographic and socioeconomic data and questions were taken from the Hungarian version of the Health Behavior in School-Aged Children (HBSC) survey (40). We used the following demographic and socioeconomic data: gender, the parents' education level (maximum primary school, vocational certificate, secondary/high school, university, or college), and the participant's family affluence. We measured family affluence with the Family Affluence Scale (FAS III), which can be used to assess material assets in a family. A previous article describes the FAS III scale in detail (41).

#### Measurement of Health-Related Knowledge

The research team developed most of the health-related knowledge tests to gauge students' knowledge. The health-related knowledge test covered topics from the intervention sessions and from the health behavior questionnaire: nutrition, physical activity, risky behavior (alcohol consumption, smoking, and addiction), and sexuality. We calculated the students' average score for each topic. Overall, a higher score implied better knowledge. During the analysis, we examined the change in test scores overall and by topic. The highest score on the nutrition knowledge test was 31 (Cronbach's alpha = 0.59); in the physical activity section it was 34 (Cronbach's alpha = 0.79); in the risky behavior section it was 27 (Cronbach's alpha = 0.71); and in the sexuality section it was 52 (Cronbach's alpha = 0.78). For the total health-related knowledge test, the maximum score was 145 (Cronbach's alpha = 0.90).

#### Measurement of Health Behavior

To compile the health behavior questionnaire, we used questions from three previous nationwide studies in Hungary after preliminary consultation with the research leaders. Thus, we developed our questionnaire based on the self-administered, anonymous questionnaire employed in the Hungarian Health Behavior in School-Aged Children 2014 (HBSC 2014) survey (40), the School Health Communication Survey (42), and the Hungarian European School Survey Project on Alcohol and Other Drugs 2015 (ESPAD 2015) (43).

We measured the frequency of breakfast consumption; students were asked how often they had breakfast on weekdays. For analysis, we dichotomized the variable: breakfast on 5 weekdays or less (40). We determined healthy and unhealthy eating through scales. We assessed how often students consumed vegetables, fruits, sweets, sugary soft drinks, energy drinks, salty snacks, and fast food (40, 44). We formed two scales from the variables: “healthy eating scale” and “unhealthy eating scale.” To compile the scales, we first converted the answers into numerical values, as follows: never = 0, less than once a week = 0.25, once a week = 1, 2–4 days a week = 3, 5–6 days a week = 5.5, and at least once a day = 7. Subsequently, we summed the scores for fruit and vegetable consumption to obtain a “healthy eating” scale ranging from 0 to 14. For the “unhealthy eating scale,” we added scores for sweets, sugary soft drinks, energy drinks, salty crisps, and fast food consumption. The scale ranged from 0 to 35. A higher score on the scales indicates more frequent consumption of healthy or unhealthy foods (45, 46).

We established the students' moderate-to-vigorous physical activity (MVPA) and vigorous physical activity (VPA) using the HBSC 2014 methodology (40, 47). For analysis, we dichotomized the MVPA variable based on the literature; the cutoff point was exercise performed for 5 days or longer (48). For the data analyses, we dichotomized the answer to the VPA question; the cutoff point was being active at least 2 times per week (40, 47).

We defined screen time as including watching TV, videos, or DVDs; playing games on a computer or game console; and using a computer for email, the internet, or homework (40, 47). For analysis, we dichotomized the variable; the cutoff point was 4 h or more on weekdays (49).

In connection with smoking habits, we examined the frequency of regular and electronic cigarette use (40, 43). For the analysis of our intervention's effectiveness, we dichotomized the variables of smoking, in which we considered non-smokers to be “never” smokers (40, 50).

We measured the monthly prevalence of alcohol consumption, drunkenness, and binge drinking before completing the questionnaire. A student engaged in binge drinking if he/she consumed 5 or more units of alcohol at one time (a unit of alcohol = 250 ml of beer, 100 ml of wine, 60 ml of vermouth/liqueur, or 30 ml of a short drink) (40, 43).

We also looked at the lifetime prevalence of sexual intercourse and the usage of condoms, contraceptive pills, and other contraceptive methods during the participant's last experience of intercourse (40).

We also examined the students' self-rated health (40) using a dichotomous variable of the self-rated health indicator to analyze our intervention's effectiveness; one category was rated by those who thought their health was excellent or good, while the other category was rated by those who viewed their health as fair, bad, or very bad.

We used 5-item questions to gauge students' attitudes toward a healthy lifestyle. Students were asked to answer:

“How important is it to you…”

- “what is healthy and what is not?”
- “to avoid unhealthy items like foods that are too fat or high in sugar?”
- “to drink sugar-free soft drinks rather than sugary drinks?”
- “to use a computer, play electronic games, watch TV?”
- “to move, play sports, exercise?”

They had to mark their answers to the questions on a 5-point scale: 1: “not important at all,” 2: “not important,” 3: “maybe important,” 4: “important,” and 5: “very important” (42). From the answers to the five questions, we created a new scale. To create the scale, first we reversed the value of the answers to the question “How important is it to you to use a computer, play electronic games, or watch TV?”. So the answer “not important at all” was worth 5 points, and the answer “very important” was worth 1 point. With this modification, we added the value of the answers to the questions to form a healthy lifestyle attitudes scale of 5–25 points (Cronbach's alpha = 0.72). A higher value on the scale indicated a more favorable attitude toward a healthy lifestyle.

#### Measurement of Nutritional Status

Based on the Hungarian National Student Fitness Test (NETFIT^®^) (51), we collected data on students' body fat percentage, height, and weight to calculate their BMI (52).

### Data Processing and Statistical Analyses

At baseline, we included 145 adolescents in the intervention group and 115 respondents in the control group. We excluded 68 students from the analysis because (1) these students had to repeat a year due to their unsatisfactory academic performance and that's why they interchanged between the intervention and control groups or have duplicated baseline data (*n* = 17), (2) they did not participate in the baseline or post-intervention surveys due to their persistent absence from school at the time of completing the questionnaire (*n* = 51). The restriction of this sample resulted in a database of 192 people with baseline and post-intervention data.

We used descriptive univariate analyses to describe the baseline characteristics of the adolescents. After taking random missing patterns into consideration, we imputed all variables that contained missing values with multiple imputation through fully conditional specification, which is an iterative Markov chain Monte Carlo (MCMC) method.

We quantified the intervention's effect on the imputed database using generalized estimating equations (GEE), which comprise a generalized linear modeling technique for modeling longitudinal correlated or clustered data. We assessed issues related to breakfast, exercise frequency, screen time, cigarette use, e-cigarette use, last month's alcohol consumption, drunkenness, binge drinking, sexual intercourse, contraception use during one's last experience of intercourse, and self-rated health via logistic models. We expressed the results using odds ratios (ORs) and their associated 95% confidence intervals (CIs). We normalized knowledge test scores, healthy and unhealthy eating and attitude scores, and BMI and body fat percentage scores through Cox-Box transformation, then tested them in linear models as continuous variables. We described the relationships between dependent and independent variables using regression coefficients (β) and their associated 95% CIs. We employed the IBM SPSS 25.0 software package for the analyses.

To determine the intervention-specific effect, we took into account the differences between baseline and post-intervention status of the participants and differences between the intervention and control groups, where the question was whether the change in the outcome from pre-intervention to post-intervention differed between the members of two groups (intervention and control). We measured this directly *via* the interaction of the intervention and the time period they spent in the study in the GEE models.

We corrected all analyses for the respondents' gender and family affluence, their parents' education level, and the time period they spent in the study (2 or 3 years). To assess the intervention's effectiveness in all models, we also examined the differences between the baseline and post-intervention survey results and between the intervention and control groups using the individual follow-up data. The results of these analyses were summarized in tables, where the “specific effect of the intervention” means the main result after adjusting for the above mentioned factors. The conclusions about the effect of the intervention were drawn based on these values.

## Results

We scrutinized data from 192 students after we cleaned the data: 43.8% at the 3-year follow-up and 56.2% at the 2-year follow-up. More than half of the students belonged to the intervention group; 49.5% were boys, 44.3% were high school students, 24.5% were vocational high school students, and 34.2% were vocational school students (Table 2).

**Table 2:** Percentage of students who participated in both the baseline and end-of-study surveys according to their main characteristics (*n* = 192).

		***n***	**%**
Time spent in the	3-year study period (involved in 2016)	84	43.8
study	2-year study period (involved in 2017)	108	56.2
Study groups	Control group	84	43.8
	Intervention group	108	56.2
Students' gender	Boy	95	49.5
	Girl	97	50.5
Type of educational	High school	85	44.3
institution	Vocational high school	47	24.5
	Secondary school	60	31.2

Most fathers had a lower level of education than mothers: a higher proportion of fathers had a vocational certificate or lower, while more mothers had a baccalaureate or diploma. Nearly half of the students had a low FAS, one-third had medium FAS, and only one-fifth had high family affluence.

We found a positive relationship between the intervention's specific effect and the risky behavior, sexuality, and total knowledge test scores (Table 3).

**Table 3:** Determinants of students' scores on the knowledge test and evaluation of the intervention's specific effect based on multivariate analysis.

		**Total knowledge test score** **β [95% CI]**	**Nutrition knowledge test score** **β [95% CI]**	**Physical activity knowledge test score** **β [95% CI]**	**Risky behavior knowledge test score** **β [95% CI]**	**Sexuality knowledge test score** **β [95% CI]**	**Healthy lifestyle attitude scale** **β [95% CI]**
Students' gender (ref.: boy)	Girl	**9.80 [5.50; 14.10]**	**1.98 [0.83; 3.13]**	1.07 [−0.17; 2.32]	**2.00 [1.04; 2.96]**	**4.03 [2.28; 5.77]**	−0.47 [−1.23; 0.29]
Father's education level (ref.: primary or less)	University or college degree	11.64 [−1.87; 25.15]	**4.09 [0.76; 7.43]**	1.64 [−2.16; 5.44]	1.84 [−0.58; 4.26]	3.59 [−2.72; 9.90]	−0.19 [−1.87; 1.49]
	Secondary school/high school	**10.04 [2.14; 17.94]**	**2.42 [0.14; 4.70]**	1.89 [−0.31; 4.09]	**2.83 [1.07; 4.58]**	**3.58 [0.47; 6.69]**	0.33 [−0.78; 1.44]
	Vocational school	**7.17 [1.04; 13.31]**	1.30 [−0.29; 2.88]	**1.78 [0.20; 3.36]**	**2.29 [0.88; 3.71]**	2.0 [−0.71; 4.72]	0.33 [−0.56; 1.23]
Mother's education level (ref.: primary or less)	University or college degree	7.76 [−1.76; 17.27]	−0.52 [−2.97; 1.92]	2.53 [−0.11; 5.17]	**2.60 [0.77; 4.42]**	2.07 [−1.94; 6.08]	0.75 [−0.53; 2.01]
	Secondary school/high school	1.65 [−5.48; 8.77]	−0.34 [−2.08; 1.40]	1.14 [−0.80; 3.09]	0.49 [−1.10; 2.07]	0.80 [−1.85; 3.45]	**1.27 [0.27; 2.26]**
	Vocational school	3.27 [−2.73; 9.28]	0.19 [−1.37; 1.75]	0.74 [−0.93; 2.41]	0.64 [−0.66; 1.95]	1.35 [−1.04; 3.74]	0.64 [−0.28; 1.55]
Family affluence (ref.: low)	High	−1.40 [−5.88; 3.09]	−0.61 [−1.78; 0.55]	−0.40 [−1.77; 0.97]	−0.41 [−1.67; 0.85]	−0.68 [−2.67; 1.30]	0.617 [−0.41; 1.64]
	Medium	−0.93 [−4.52; 2.66]	0.16 [−0.75; 1.07]	−0.32 [−1.34; 0.70]	−0.26 [−1.19; 0.67]	−0.54 [−2.17; 1.08]	0.45 [−0.32; 1.22]
Time spent in the study (ref.: 2 years)	3 years	−2.04 [−6.45; 2.37]	−0.59 [−1.72; 0.54]	−0.80 [−2.01; 0.41]	−0.82 [−1.81; 0.17]	0.117 [−1.74; 1.97]	−0.25 [−0.96; 0.47]
End-of-study survey (ref.: baseline survey)		0.17 [−2,82; 3,16]	**0.96 [0.15; 1.78]**	−1.15 [−2.37; 0.07]	**0.95 [0.14; 1.76]**	−0.328 [−1.79; 1.13]	−0.32 [−1.00; 0.36]
Intervention group (ref.: control group)		−1.48 [−5,26; 2,31]	−0.42 [−1.64; 0.80]	−0.16 [−1.48; 1.16]	−0.54 [−1.54; 0.47]	−0.19 [−1.823; 1.44]	0.19 [−0.67; 1.05]
The intervention's specific effect (interaction of the intervention and the time period they spent in the intervention)		**7.84 [3, 53; 12, 15]**	0.41 [−0.73; 1.55]	0.69 [−0.83; 2.19]	**1.74 [0.57; 2.91]**	**4.07 [2.02; 6.13]**	−0.74 [−1.70; 0.22]

*β, regression coefficient calculated from a generalized estimation equation; 95% CI, 95% confidence interval; in bold, significant associations*.

A further outcome of our intervention was that among the students in the intervention group, the consumption of unhealthy foods was significantly lower (Table 4), and the frequency of exercise significantly increased (Table 5). There was a significant decline in the number of students in the intervention group who spent <3 h playing on computers or consoles (Table 5). There was a significantly greater chance of alcohol abstinence by the end of the study. In addition, the chances of not smoking also increased, but this was not significant (Table 6).

**Table 4:** Determinants of students' eating habits and evaluation of the intervention's specific effect based on multivariate analysis.

		**Scale of healthy eating** **β [95% CI]**	**Scale of unhealthy eating** **β [95% CI]**	**Breakfast on weekdays (5 times)** **OR [95% CI]**
Students' gender (ref.: boy)	Girl	0.57 [−0.41; 1.55]	−0.12 [−2.16; 1.91]	0.71 [0.41; 1.24]
Father's education level (ref.: primary or less)	University or college degree	−1.50 [−4.38; 1.37]	1.09 [−4.20; 6.39]	0.32 [0.07; 1.42]
	Secondary school/high school	−0.04 [−1.67; 1.59]	−0.61 [−4.59; 3.37]	0.44 [0.17; 1.13]
	Vocational school	0.13 [−1.29; 1.55]	−0.21 [−3.57; 3.15]	**0.41 [0.19; 0.88]**
Mother's education level (ref.: primary or less)	University or college degree	0.09 [−2.00; 2.18]	**−6.75 [−11.35;** **−2.15]**	2.45 [0.78; 7.69]
	Secondary school/high school	0.35 [−1.13; 1.82]	**−6.20 [−9.07;** **−3.33]**	1.46 [0.66; 3.25]
	Vocational school	−0.41 [−1.74; 0.92]	**−6.28 [−9.15;** **−3.42]**	1.10 [0.51; 2.33]
Family affluence (ref.: low)	High	0.44 [−0.81; 1.70]	−1.37 [−3.75; 1.01]	0.72 [0.38; 1.34]
	Medium	0.31 [−0.53; 1.15]	−1.32 [−2.87; 0.23]	1.04 [0.65; 1.69]
Time spent in the study (ref.: 2 years)	3 years	0.50 [−0.40; 1.39]	−0.67 [−2.80; 1.47]	0.61 [0.36; 1.04]
End-of-study survey (ref.: baseline survey)		−0.55 [−1.46; 0.37]	1.50 [−0.27; 3.27]	0.78 [0.44; 1.39]
Intervention group (ref.: control group)		−0.03 [−1.05; 1.00]	1.29 [−0.98; 3.55]	1.26 [0.68; 2.34]
The intervention's specific effect (interaction of the intervention and the time period they spent in the intervention)		0.02 [−1.20; 1.24]	**−2.78 [−5.02;** **−0.54]**	0.79 [0.38; 1.61]

*β, regression coefficient calculated from a generalized estimation equation; 95% CI, 95% confidence interval; in bold, significant associations*.

**Table 5:** Determinants of students' physical activity and screen time, and evaluation of the intervention's specific effect based on multivariate analysis.

		**Report at least 60 min of MVPA (5 or more days a week)** **OR [95% CI]**	**Report VPA at least twice a week** **OR [95% CI]**	**Watch television, videos, or DVDs for 3 or more hours on weekdays** **OR [95% CI]**	**Playing games on a computer or game console for 3 or more hours on weekdays** **OR [95% CI]**	**Using a computer for email, the internet, or homework for 3 or more hours on weekdays** **OR [95% CI]**
Students' gender (ref.: boy)	Girl	**0.28 [0.16; 0.48]**	**0.25 [0.15; 0.43]**	0.95 [0.50; 1.80]	**2.33 [1.14; 4.77]**	1.00 [0.59; 1.69]
Father's education level (ref.: primary or less)	University or college degree	1.20 [0.31; 4.60]	3.43 [0.63; 18.61]	2.03 [0.39; 10.45]	6.01 [0.68; 52.96]	1.05 [0.20; 5.43]
	Secondary school/high school	1.25 [0.48; 3.26]	1.45 [0.55; 3.81]	1.42 [0.53; 3.79]	1.06 [0.33; 3.45]	0.45 [0.17; 1.22]
	Vocational school	1.42 [0.66; 3.04]	1.77 [0.82; 3.82]	0.99 [0.48; 2.06]	1.08 [0.39; 3.02]	0.58 [0.24; 1.42]
Mother's education level (ref.: primary or less)	University or college degree	2.73 [0.91; 8.25]	1.09 [0.33; 3.60]	1.91 [0.52; 7.09]	1.90 [0.59; 6.10]	**6.00 [2.02; 17.83]**
	Secondary school/high school	1.19 [0.53; 2.69]	0.89 [0.40; 1.95]	1.30 [0.65; 2.59]	1.84 [0.74; 4.59]	**3.00 [1.39; 6.48]**
	Vocational school	0.86 [0.37; 1.98]	0.85 [0.42; 1.75]	**2.46 [1.10; 5.48]**	**6.34 [2.01; 19.93]**	**3.07 [1.33; 7.09]**
Family affluence (ref.: low)	High	1.15 [0.59; 2.26]	1.91 [0.97; 3.77]	0.82 [0.38; 1.75]	1.09 [0.52; 2.31]	0.66 [0.34; 1.28]
	Medium	1.26 [0.74; 2.16]	**1.77 [1.08; 2.92]**	0.89 [0.49; 1.64]	1.55 [0.74; 3.22]	0.83 [0.47; 1.47]
Time spent in the study (ref.: 2 years)	3 years	0.95 [0.55; 1.66]	0.77 [0.44; 1.33]	0.91 [0.50; 1.66]	0.98 [0.50; 1.90]	1.05 [0.62; 1.77]
End-of-study survey (ref.: baseline survey)		**0.30 [0.16; 0.55]**	0.66 [0.41; 1.06]	1.26 [0.64; 2.47]	**2.51 [1.14; 5.54]**	0.95 [0.51; 1.79]
Intervention group (ref.: control group)		1.01 [0.54; 1.89]	1.07 [0.55; 2.08]	1.39 [0.65; 2.98]	2.41 [1.00; 5.82]	0.80 [0.40; 1.59]
The intervention's specific effect (interaction of the intervention and the time period they spent in the intervention)		**2.19 [1.01; 4.76]**	1.06 [0.52; 2.15]	0.59 [0.23; 1.47]	**0.27 [0.09; 0.79]**	0.93 [0.41; 2.14]

*OR, odds ratio calculated from a generalized estimation equation; 95% CI, 95% confidence interval; MVPA, moderate-to-vigorous physical activity; VPA, vigorous physical activity; in bold, significant associations*.

**Table 6:** Determinants of students' smoking and alcohol consumption habits, and evaluation of the intervention's specific effect based on multivariate analysis.

		**Do not use cigarettes** **OR [95% CI]**	**Do not use e-cigarettes** **OR [95% CI]**	**Have not drunk alcohol in the last 30 days** **OR [95% CI]**	**Have not been drunk in the last 30 days** **OR [95% CI]**	**Have not been a binge drinker in the last 30 days** **OR [95% CI]**
Students' gender (ref.: boy)	Girl	1.64 [0.92; 2.93]	**2.49 [1.34; 4.62]**	1.57 [0.94; 2.62]	**2.08 [1.21; 3.60]**	**2.15 [1.28; 3.61]**
Father's education level (ref.: primary or less)	University or college degree	1.25 [0.24; 6.63]	0.25 [0.05; 1.45]	0.57 [0.14; 2.35]	0.88 [0.20; 3.91]	2.44 [0.68; 8.73]
	Secondary school/high school	0.87 [0.34; 2.29]	**0.31 [0.11; 0.89]**	0.86 [0.35; 2.13]	0.47 [0.20; 1.10]	1.11 [0.44; 2.79]
	Vocational school	0.90 [0.42; 1.96]	0.57 [0.24; 1.36]	0.71 [0.34; 1.50]	0.66 [0.32; 1.34]	1.33 [0.63; 2.77]
Mother's education level (ref.: primary or less)	University or college degree	1.80 [0.56; 5.83]	2.56 [0.60; 10.97]	1.78 [0.60; 5.27]	2.53 [0.84; 7.67]	1.73 [0.61; 4.92]
	Secondary school/high school	1.58 [0.74; 3.38]	1.76 [0.77; 4.02]	1.57 [0.74; 3.33]	2.08 [0.99; 4.34]	1.14 [0.55; 2.36]
	Vocational school	1.48 [0.73; 3.00]	1.41 [0.63; 3.13]	0.89 [0.43; 1.84]	1.47 [0.75; 2.88]	0.92 [0.46; 1.83]
Family affluence (ref.: low)	High	1.34 [0.76; 2.37]	1.63 [0.73; 3.64]	0.68 [0.36; 1.29]	0.62 [0.33; 1.20]	0.74 [0.42; 1.33]
	Medium	1.16 [0.71; 1.9]	1.16 [0.63; 2.13]	1.27 [0.78; 2.09]	0.82 [0.48; 1.40]	1.29 [0.76; 2.17]
Time spent in the study (ref.: 2 years)	3 years	0.80 [0.45; 1.41]	0.72 [0.39; 1.32]	**0.59 [0.36; 0.99]**	0.62 [0.36; 1.06]	0.62 [0.37; 1.06]
End-of-study survey (ref.: baseline survey)		**0.20 [0.12; 0.34]**	**0.23 [0.11; 0.48]**	**0.25 [0.14; 0.44]**	**0.44 [0.24; 0.81]**	**0.42 [0.24; 0.71]**
Intervention group (ref.: control group)		0.86 [0.42; 1.78]	1.26 [0.45; 3.49]	0.72 [0.40; 1.30]	1.52 [0.70; 3.30]	1.59 [0.80; 3.15]
The intervention's specific effect (interaction of the intervention and the time period they spent in the intervention)		1.77 [0.92; 3.39]	1.08 [0.37; 3.14]	**2.12 [1.01; 4.43]**	0.56 [0.25; 1.28]	0.59 [0.28; 1.22]

*OR, odds ratio calculated from a generalized estimation equation; 95% CI, 95% confidence interval; in bold, significant associations*.

Our intervention did not affect students' healthy lifestyle attitudes (Table 3), sexual behavior (Table 7), self-rated health, or nutritional status (Table 8).

**Table 7:** Determinants of students' sexual behavior, and evaluation of the intervention's specific effect based on multivariate analysis.

		**Have not had sexual intercourse** **OR [95% CI]**	**Used a condom, contraceptive pill, or other contraceptive method during the last experience of intercourse** **OR [95% CI]**
Students' gender (ref.: boy)	Girl	1.66 [0.95; 2.91]	1.20 [0.49; 2.94]
Father's education level (ref.: primary or less)	University or college degree	0.51 [0.12; 2.21]	2.78 [0.29; 26.41]
	Secondary school/high school	0.84 [0.33; 2.13]	1.50 [0.36; 6.32]
	Vocational school	0.85 [0.40; 1.78]	1.97 [0.56; 6.89]
Mother's education level (ref.: primary or less)	University or college degree	2.28 [0.73; 7.18]	0.89 [0.12; 6.47]
	Secondary school/high school	1.67 [0.75; 3.69]	1.49 [0.35; 6.36]
	Vocational school	2.02 [0.95; 4.30]	0.61 [0.17; 2.14]
Family affluence (ref.: low)	High	0.70 [0.35; 1.40]	1.71 [0.54; 5.48]
	Medium	0.95 [0.56; 1.60]	1.20 [0.44; 3.31]
Time spent in the study (ref.: 2 years)	3 years	**0.33 [0.19; 0.59]**	1.54 [0.58; 4.06]
End-of-study survey (ref.: baseline survey)		**0.20 [0.11; 0.34]**	0.23 [0.02; 2.62]
Intervention group (ref.: control group)		1.49 [0.69; 3.24]	0.10 [0.01; 1.21]
The intervention's specific effect (interaction of the intervention and the time period they spent in the intervention)		0.582 [0.261; 1.30]	6.97 [0.43; 111.94]

*OR, odds ratio calculated from a generalized estimation equation; 95% CI, 95% confidence interval; in bold, significant associations*.

**Table 8:** Determinants of students' self-rated health and nutritional status, and evaluation of the intervention's specific effect based on multivariate analysis.

		**Self-rated health is good or excellent** **OR [95% CI]**	**BMI** **β [95% CI]**	**Body fat percentage** **β [95% CI]**
Students' gender (ref.: boy)	Girl	**0.40 [0.24; 0.67]**	−0.67 [−1.97; 0.63]	**10.07 [7.92; 12.23]**
Father's education level (ref.: primary or less)	University or college degree	1.06 [0.34; 3.26]	−1.49 [−4.59; 1.60]	−4.19 [−11.00; 2.63]
	Secondary school/high school	1.16 [0.48; 2.82]	**−2.32 [−4.52;** **−0.13]**	**−4.65 [−8.13;** **−1.17]**
	Vocational school	0.86 [0.41; 1.80]	−1.25 [−2.97; 0.47]	−2.75 [−5.61; 0.11]
Mother's education level (ref.: primary or less)	University or college degree	1.04 [0.39; 2.76]	1.93 [−0.17; 4.04]	1.05 [−4.46; 6.55]
	Secondary school/high school	0.93 [0.46; 1.87]	1.37 [−0.20; 2.95]	2.42 [−0.62; 5.47]
	Vocational school	0.64 [0.32; 1.25]	1.56 [−0.08; 3.19]	1.98 [−0.62; 4.58]
Family affluenc e (ref.: low)	High	1.00 [0.56; 1.79]	**1.18 [0.14; 2.21]**	2.15 [−0.70; 5.00]
	Medium	0.91 [0.55; 1.52]	0.22 [−0.49; 0.92]	−0.80 [−2.42; 0.82]
Time spent in the study (ref.: 2 years)	3 years	0.80 [0.49; 1.31]	0.29 [−1.00; 1.57]	−0.42 [−2.58; 1.74]
End-of-study survey (ref.: baseline survey)		1.28 [0.74; 2.22]	**0.79 [0.06; 1.52]**	**2.09 [0.68; 3.49]**
Intervention group (ref.: control group)		1.56 [0.84; 2.91]	0.07 [−1.31; 1.45]	1.30 [−1.04; 3.65]
The intervention's specific effect (interaction of the intervention and the time period they spent in the intervention)		0.74 [0.37; 1.50]	−0.21 [−1.22; 0.80]	−0.88 [−3.03; 1.28]

*β, regression coefficient calculated from the generalized estimation equation; OR, odds ratio; calculated from the generalization estimation equation; 95% CI, 95% confidence interval; in bold, significant associations*.

## Discussion

Our high school intervention program, which focuses on the development of knowledge and skills and covers several segments of a healthy lifestyle, was embedded into the curriculum in the framework of the school system and school health promotion. As a result of the intervention study, it became possible to improve the students' knowledge about health, as well as some areas of their health behavior.

Students' overall knowledge in the intervention group showed a significant increase compared to the control group, except for the tests regarding diet and physical activity. Both the intervention and the control group included students—among others—from the food industry (bakers, confectioners, and chefs), trade, hospitality, and sports areas for which training contains more information about nutrition and physical activity. This might be the reason that the additional knowledge that could be gained from the intervention couldn't be detected regarding these topics. Appropriate knowledge is a necessary condition for behavior change but alone is not enough (e.g., the phenomenon of cognitive dissonance).

Our intervention was not able to change the students' attitudes toward a healthy lifestyle in a positive direction. According to the Theory of Planned Behavior attitude is one of the determinants of the person's intention, which represents his/her motivation. Within this theory, intention to engage in a certain behavior together with perceived behavior control will determine the person's behavior (53).

Our intervention had a positive impact on unhealthy diet; however, we did not achieve a positive shift in terms of breakfast regularity and healthy eating (fruit and vegetable consumption) compared to the control group. One reason may be that adolescents have much more control over the consumption of unhealthier foods than the consumption of breakfast or fruits and vegetables. The latter is perhaps more influenced by family eating habits and affluence (1, 54, 55). The students' frequency of MVPA increased compared to that of the control group. In the intervention group, the duration of playing games on a computer or game console rose significantly. The Hungarian HBSC 2018 survey examined the relationship between physical activity and screen time, and found that not only those who do not move in their free time spend more time playing computer games, but also those who exercise daily; the relationship between screen time and exercise turned out to be U-shaped (50). In this way, our findings are in line with the Hungarian national experience.

Regarding cigarette use, there was a more favorable trend in the intervention group than in the control group, but this trend was not statistically significant. This outcome is encouraging, as it is conceivable that there would have been a significant difference between the two groups if the intervention were to have been continued. Our intervention also had a positive effect on the frequency of alcohol consumption, and last month's abstinence was significantly more likely in the intervention group.

Our intervention failed to influence sexual behavior (sexual activity and contraception use), self-rated health, and nutritional status. The latter may be due to positive shift in health status where nutrition might be expected in the longer term following a change in health behavior. Improving these indicators could be facilitated by health promotion programs that focus more on students' physical activity (56) and eating habits (e.g., by transforming the school environment to encourage exercise and healthy eating, or by promoting healthier eating opportunities through peer helpers) (57).

### Strengths and Limitations

#### Planning

One of the limitations of our intervention is that the intervention and control group studied in the same school, so we cannot rule out that the knowledge and skills acquired during the intervention may have also appeared among the members of the control group. But taking into account that it can lead to the underestimation of the impact of our intervention, this could not jeopardize our conclusions. Due to the design of the study, we could not change the wider school environment (e.g., using posters, courtyards, stair decorations), which could have had a further positive effect on students' health behavior. However, from a research point of view, the difference between the intervention and control groups was probably smaller in light of other background factors that could potentially influence the effect of the intervention (e.g., exercise opportunities in the broader environment, food supply) than in the case of another control group.

One of the study's strengths is that we took into account the aspects of IUHPE (19), namely, that our program has been operating at regular intervals for several years and addressing several segments of a healthy lifestyle.

#### Implementation

The goal of the program was hampered by the COVID-19 pandemic, which resulted in restrictions for the spring of 2020, when some sessions and the closing data collection had to be canceled. This may have had a negative effect on the results obtained, and we can assume that based on the data for the entire time period, changes in other areas could be made as well.

From the angle of IUHPE (19), we were able to involve the health sector in our program and to coordinate some parts of our work with the activities of the local health promotion office.

A further strength of the program was that, as part of the intervention, a public health professional participated in the daily life of the school and regularly consulted with the school management and teachers regarding the planned intervention sessions and measurements. Thanks to this individual's presence, the teachers became more open to the program, and some teachers volunteered for a certain activity. Professional participation in school life also helped to facilitate the students' acceptance of the program and to build a solid relationship with them, which was essential for effective collaboration.

Another strength of the program is that, with cost-effectiveness in mind, we tried to develop it taking into account the school's capabilities, so we mostly used existing and easily accessible tools (e.g., blackboard, computer, projector, websites, and telephone applications) during the intervention. The intervention sessions were carried out taking into consideration the specifics of each class (e.g., prior knowledge, ability), which promoted equal opportunities to acquire knowledge, favorable attitudes, and health behavior. Further, students were able to access useful websites and telephone applications that provided reliable information learned and practiced during the sessions, regardless of space, people, or time outside of school, thus providing safe guidance for developing appropriate health behaviors. Given the elements of the program that have taken place, we can assume that, with the coordination of a public health professional or trained teachers, our program can be used in other schools based on the capabilities of each school and its students. However, when planning and implementing a school health education program, it is necessary to consider the prior knowledge, abilities, and skills of the target group together with their needs and shape the planned course accordingly. But it is also important to take into consideration the possibilities and the characteristics of the setting.

#### Evaluation

On the negative side, the necessary data cleaning process due to the nature of the individual follow-up reduced the size of the already relatively small sample, which limited the study's statistical power.

There was no systematic evaluation undertaken with the students to find out how they felt about the program, partly because of the length of the questionnaire. Still, they had the opportunity to share their thoughts during the sessions.

We were unable to control all potential influencing factors, especially the impact of advertising or social media on the adolescents' health behavior was not investigated during the study.

The data analysis and evaluation of the intervention were made by an independent statistician, who was not involved in the planning or delivery of the intervention.

Within the limits of our study, in some cases, we could not identify differences due to the absence of adequate statistical power. We could not perform stratified data analysis due to the nature of the sample and the relatively low sample size. The shortcomings resulting from the low statistical power are nuanced by the fact that we corrected the statistical metrics obtained during the analysis for sociodemographic factors; therefore, the indicators show the connection between the dependent and independent variables without the effect of sociodemographic confounders.

The system used for anonymous individual tracking of students in the study (individual code provided by the student and answers to questions similar to password reminders) not only worked well for follow-up but also facilitated the anonymous use of NETFIT data and the other datasheets (i.e., health behavior questionnaire, knowledge test).

### Conclusions

Schools are cost-effective settings for health education programs and are critical areas for developing health-related knowledge of children and adolescents. Based on our experiences, the COM-B model can be used as a theoretical framework for designing complex school health promotion programs. These programs can be most successful if they not only cultivate the target group's knowledge regarding a healthy lifestyle but also its skills, thus motivating the group members (39), and—in line with the IUHPE findings—operate in a supportive social and physical environment over time (19). Therefore, in 2016, we tried to take all these factors into account when planning our school education program. For several years, our intervention study sought to shape students' knowledge, skills, attitudes, health behavior, and health in a positive direction using a variety of interactive methods and digital tools, and to monitor the effectiveness of our health promotion program at regular intervals.

Our intervention also achieved positive results in terms of knowledge transfer and, in some topics, changes in health behavior. These results are in line with the conclusion of the IUHPE (19) as school-based interventions can transmit knowledge, develop skills and support healthy choices. But we have to keep in mind that the health behavior and health status of the students are influenced by other, outside of the school factors, too.

Prior research has not identified a clear connection between nutritional knowledge and healthy eating or exercise-related knowledge and physical activity (58–60). Relatedly, in childhood and adolescence, the parent is mostly responsible for providing the food consumed and ways of spending one's free time (e.g., sports funding), so adolescents can control these behaviors the least, as opposed to smoking or alcohol consumption.

Previous studies (1, 3, 4, 6, 7, 61–63) have also shown that adolescents' demographic and socioeconomic background (gender, parents' education levels, family affluence), their social environment and the media can affect their health-related knowledge, attitudes, health behavior, and health status. Hence, we cannot ignore these factors when designing health promotion programs for school-age children. Greater involvement of parents and teachers in the health promotion program, and the creation of a supportive school environment, can greatly contribute to its success. In the spirit of a comprehensive school, health promotion programs should include all segments of a healthy lifestyle, affect all students and faculty members in the school, involve other organizations in addition to parents, and keep sustainability in mind. The Balassagyarmat Health Education Program and the Buda Region Health Program (2018–2030) are good examples from Hungary, but these programs were started after our intervention, and their effectiveness has not yet been evaluated. The Balassagyarmat Health Education Program is a complex school-based health education program with peer helpers that places special emphasis on students' own knowledge, creating an environment that promotes positive health behavior and deepens motivation (34). The Buda Region Health Program (2018–2030) aims to support and strengthen health promotion and prevention activities at the local level with the involvement of local and regional authorities, health care providers, non-governmental organizations, academia, and government. The program's main objective is to promote children's health in a complex way by creating an environment that is conducive to healthy choices, the development of health services, the improvement of children's health behaviors and education, the involvement of schools and families, and the transfer of experience (35). Due to the limiting factors described earlier, as well as the goal of making the program sustainable without external funding, it was not possible in the present research to implement such a complex intervention.

In support of complex programs, there is also a need for well-designed health promotion programs to be given more space in schools' pedagogical programs, taking into account the infrastructure, community, the school's unique features, and possibilities provided by the settlement where the school is located.

Teachers can play a key role in knowledge transfer about healthy lifestyles and in shaping students' attitudes and lifestyles in a positive direction (64). In addition to imparting knowledge about healthy lifestyles, such skills may include integrating the topic of health into individual subjects, knowing and presenting about credible sources of information, planning, and organizing topic weeks and programs for healthy lifestyles, or involving peer helpers. It is also vital to shape educators' attitudes in a positive direction, as they also have a great responsibility, since the development of students' health includes having the teacher himself/herself be credible in his/her role as a health promoter.

## Data Availability Statement

The raw data supporting the conclusions of this article will be made available by the authors, without undue reservation.

## Ethics Statement

The studies involving human participants were reviewed and approved by Hungary's Medical Research Council Scientific and Research Committee. Written informed consent to participate in this study was provided by the participants' legal guardian/next of kin.

## Author Contributions

GN-P and ÉB: conceptualization and funding acquisition. GN-P, FV, and ÉB: methodology. FV: formal analysis. GN-P: investigation and project administration. GN-P and FV: writing—original draft preparation and visualization. ÉB: writing—review and editing and supervision. All authors have read and agreed to the published version of the manuscript.

## Funding

This work was supported by the EFOP-3.6.3-VEKOP-16-2017-00009, co-financed by the EU and the European Social Fund. Writing of the manuscript was supported by GINOP-2.3.2-15-2016-00005 project. This project is co-financed by the European Union under the European Regional Development Fund. The funders had no role in the design of the study; in the collection, analyses, or interpretation of data; in the writing of the manuscript, or in the decision to publish the results.

## Conflict of Interest

The authors declare that the research was conducted in the absence of any commercial or financial relationships that could be construed as a potential conflict of interest.

## Publisher's Note

All claims expressed in this article are solely those of the authors and do not necessarily represent those of their affiliated organizations, or those of the publisher, the editors and the reviewers. Any product that may be evaluated in this article, or claim that may be made by its manufacturer, is not guaranteed or endorsed by the publisher.
